# OMeta: an ontology-based, data-driven metadata tracking system

**DOI:** 10.1186/s12859-018-2580-9

**Published:** 2019-01-07

**Authors:** Indresh Singh, Mehmet Kuscuoglu, Derek M. Harkins, Granger Sutton, Derrick E. Fouts, Karen E. Nelson

**Affiliations:** 1 J. Craig Venter Institute, 9605 Medical Center Drive, Suite 150, Rockville, MD 20850 USA; 2 J. Craig Venter Institute, 4120 Capricorn Ln, La Jolla, CA 92037 USA

**Keywords:** Metadata, GSC/BRC standards, Standards, Genomics, Ontology, MIxS, MIMS, Data deposit, Data integrity

## Abstract

**Background:**

The development of high-throughput sequencing and analysis has accelerated multi-omics studies of thousands of microbial species, metagenomes, and infectious disease pathogens. Omics studies are enabling genotype-phenotype association studies which identify genetic determinants of pathogen virulence and drug resistance, as well as phylogenetic studies designed to track the origin and spread of disease outbreaks. These omics studies are complex and often employ multiple assay technologies including genomics, metagenomics, transcriptomics, proteomics, and metabolomics. To maximize the impact of omics studies, it is essential that data be accompanied by detailed contextual metadata (e.g., specimen, spatial-temporal, phenotypic characteristics) in clear, organized, and consistent formats. Over the years, many metadata standards developed by various metadata standards initiatives have arisen; the Genomic Standards Consortium’s minimal information standards (MIxS), the GSCID/BRC Project and Sample Application Standard. Some tools exist for tracking metadata, but they do not provide event based capabilities to configure, collect, validate, and distribute metadata. To address this gap in the scientific community, an event based data-driven application, OMeta, was created that allows users to quickly configure, collect, validate, distribute, and integrate metadata.

**Results:**

A data-driven web application, OMeta, has been developed for use by researchers consisting of a browser-based interface, a command-line interface (CLI), and server-side components that provide an intuitive platform for configuring, capturing, viewing, and sharing metadata. Project and sample metadata can be set based on existing standards or based on projects goals. Recorded information includes details on the biological samples, procedures, protocols, and experimental technologies, etc. This information can be organized based on events, including sample collection, sample quantification, sequencing assay, and analysis results. OMeta enables configuration in various presentation types: checkbox, file, drop-box, ontology, and fields can be configured to use the National Center for Biomedical Ontology (NCBO), a biomedical ontology server. Furthermore, OMeta maintains a complete audit trail of all changes made by users and allows metadata export in comma separated value (CSV) format for convenient deposition of data into public databases.

**Conclusions:**

We present, OMeta, a web-based software application that is built on data-driven principles for configuring and customizing data standards, capturing, curating, and sharing metadata.

**Electronic supplementary material:**

The online version of this article (10.1186/s12859-018-2580-9) contains supplementary material, which is available to authorized users.

## Background

The development of high-throughput sequencing and analysis has accelerated multi-omics studies of thousands of microbial species, metagenomes, and infectious disease pathogens. Omics tools and technologies are enabling genotype-phenotype association studies that identify genetic determinants of pathogen virulence and drug resistance as well as phylogenetic studies designed to track the origin and spread of pathogens during disease outbreaks. These omics studies are complex and often employ multiple technologies, including genomics, metagenomics, transcriptomics, proteomics, and metabolomics. To maximize the impact of omics studies, it is essential that the data be accompanied by detailed contextual metadata (e.g., organism or environmental source of the specimen, spatial-temporal information about the specimen isolation event and phenotypic characteristics) in clear, organized, and consistent formats. Over the years, various metadata standards initiatives have developed many metadata standards. Examples include the Genomic Standards Consortium’s minimal information standards (MIxS), the Genome Sequencing consortium/Bioinformatics Resource Centers (GSCID/BRC) Project and Sample Application Standard, DMID Clinical Metadata Standards (Dugan et al., 2014), the National Institute of Allergy and Infectious Diseases (NIAID) metadata working group, NCBI’s BioSample metadata, and the Ontology of Biomedical Investigations (OBI). Unfortunately, the amount and complexity of metadata required to make sense of omics data has surpassed most researcher’s ability to manage using spreadsheets. Currently, there is no easy to use, event based enterprise-level tools to configure, collect, validate, and distribute metadata. A summary of tools and their features is described in the discussion. To address this critical need for the scientific community, we built an event based, data-driven application, OMeta, which allows users to quickly configure, collect, validate, distribute, and integrate metadata. The OMeta application was designed with data-driven principles to be responsive to metadata. It enables modifications in data standards template, fields, fields ontology, event, and validation through alterations in metadata rather than code-based changes, allowing an agile response to evolving and changing metadata standards and study goals.

### Design and implementation

OMeta was designed with the following goals:

1. Easy to configure and customize for metadata tracking based on the study design
2. Ability to configure and track metadata based on any standards
3. Support event-based metadata tracking in real-time for multi-isolate studies
4. Track the complete audit trail of changes
5. Support changing metadata tracking requirements
6. Data-driven dynamic application to support evolving metadata and study
7. Easy to use

### Architecture

**Fig. 1:**
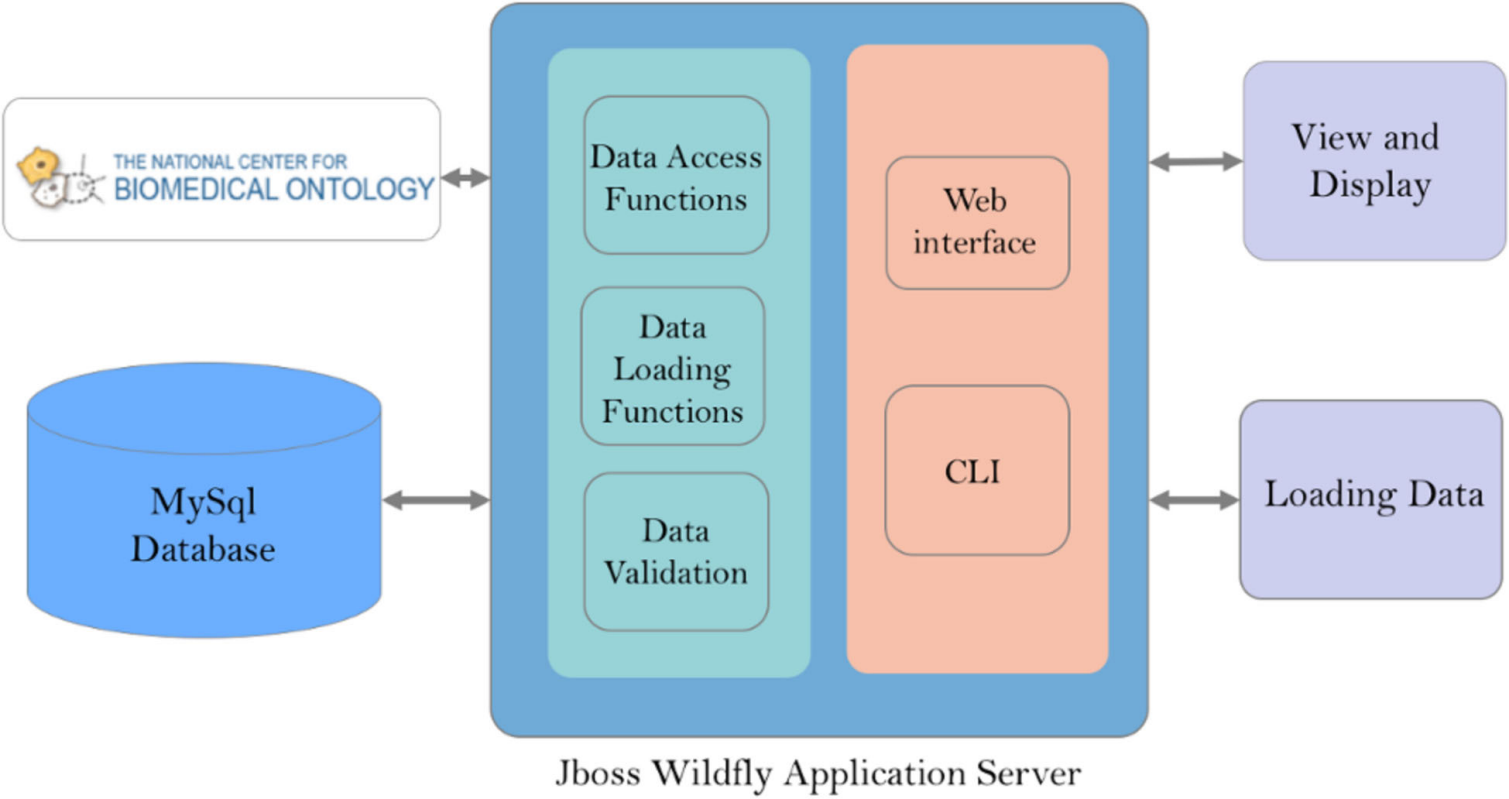
OMeta System Architecture. This diagram summarizes the system architecture. All high-level components that are part of application are represented; the NCBO ontology server, CLI, back-end MySQL database, as well as the application server with its data loading, validation, and data access modules

OMeta is an open source tool built on an open source infrastructure (Fig. 1). OMeta uses MySQL as the backend database, JBoss Wildfly as an application and web server, OpenLDAP for user authentication, and HTML/JavaScript is employed for a front-end web interface. OMeta is platform-independent and can be deployed on Windows, Linux or MacOS. OMeta presents a unique data-driven architecture that enables the application to be quickly configured with minor code changes.

### Project, sample, and events

**Fig. 2:**
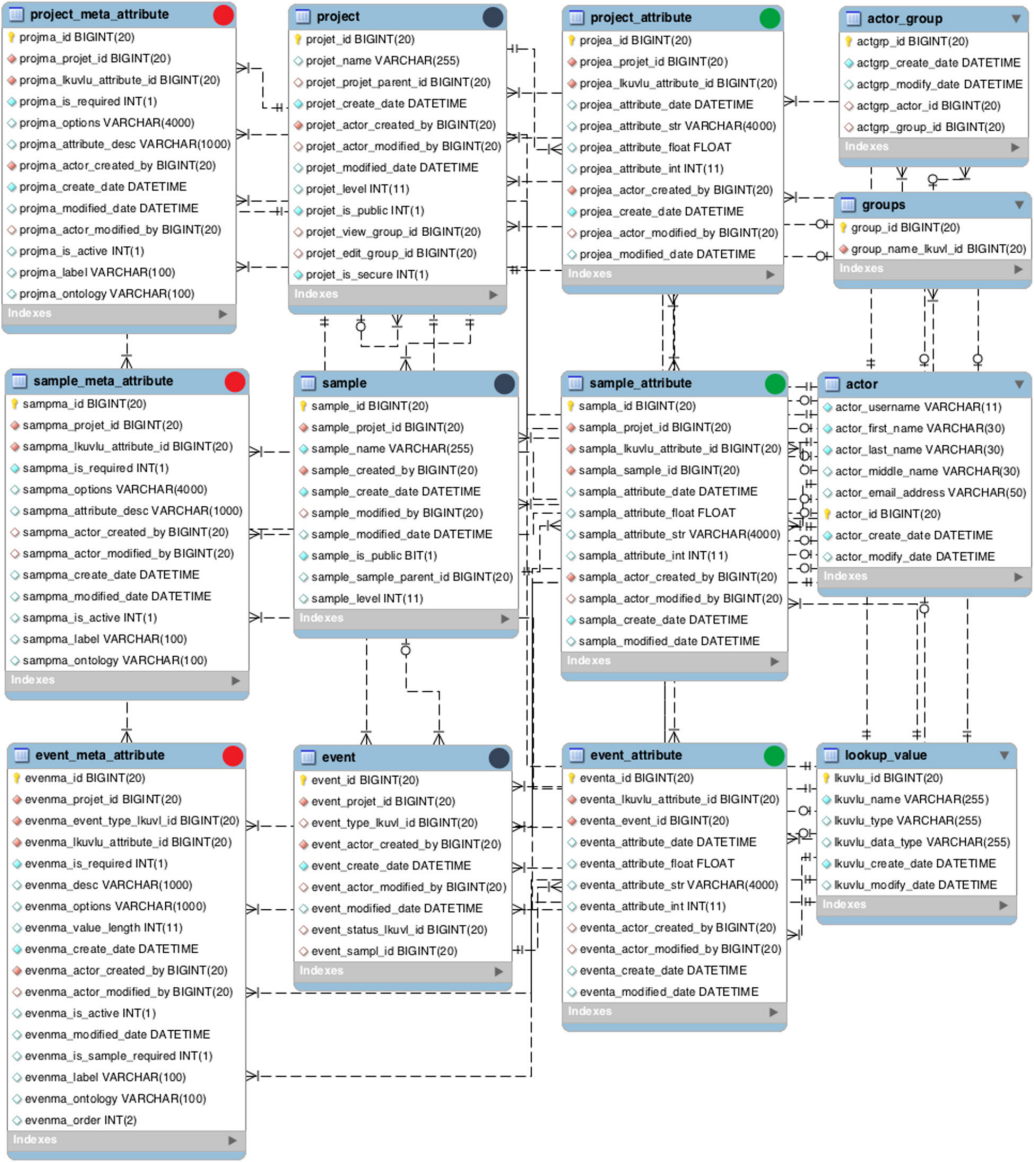
OMeta Database Schema. Metadata data tables are marked with red circles. Core data tables are marked with grey circles. Data tables are marked with green circles

OMeta’s schema is designed on three key core entities; ***Project***, ***Sample,*** and ***Event*** (Fig. 2). A ***Project*** is a high-level entity that can be a project (or study) with high level information. Examples include the Human Microbiome Project (U54AI084844), the NIAID-funded JCVI Genomic Centers for Infectious Diseases (GCID) (U19AI110819) and an NIH-sponsored oral microbiome project recently undertaken by the JCVI (R01DE019665), described below under Case Studies. A ***Sample*** is an entity representing a specific sample. It can be a biological sample, assay, reagent, or any entity that can be tracked under the project. An ***Event*** is an entity storing any event or operation that can be performed on a sample or project entity. An *Event* allows fields to be logically grouped by the process or operation, facilitating metadata views of only relevant fields. Examples of an *Event* are: project registration, project update, sample registration, sample update, sample aliquot, library preparation, sequencing status, analysis status, sequencing assay, and analysis result. OMeta has certain key events such as project registration, project update, sample registration, and sample update, but users can create new events based on study design and tracking requirements.

### Data-driven design

OMeta schema is designed based on data-driven principles [1]. In data-driven design, application functionality and behavior are driven by data, rather than hard-coded specific use-cases. We have designed OMeta to follow these data-driven principles, providing extreme flexibility and agility and allowing applications to be easily customized without modifying any underlying code.

**Fig. 3:**
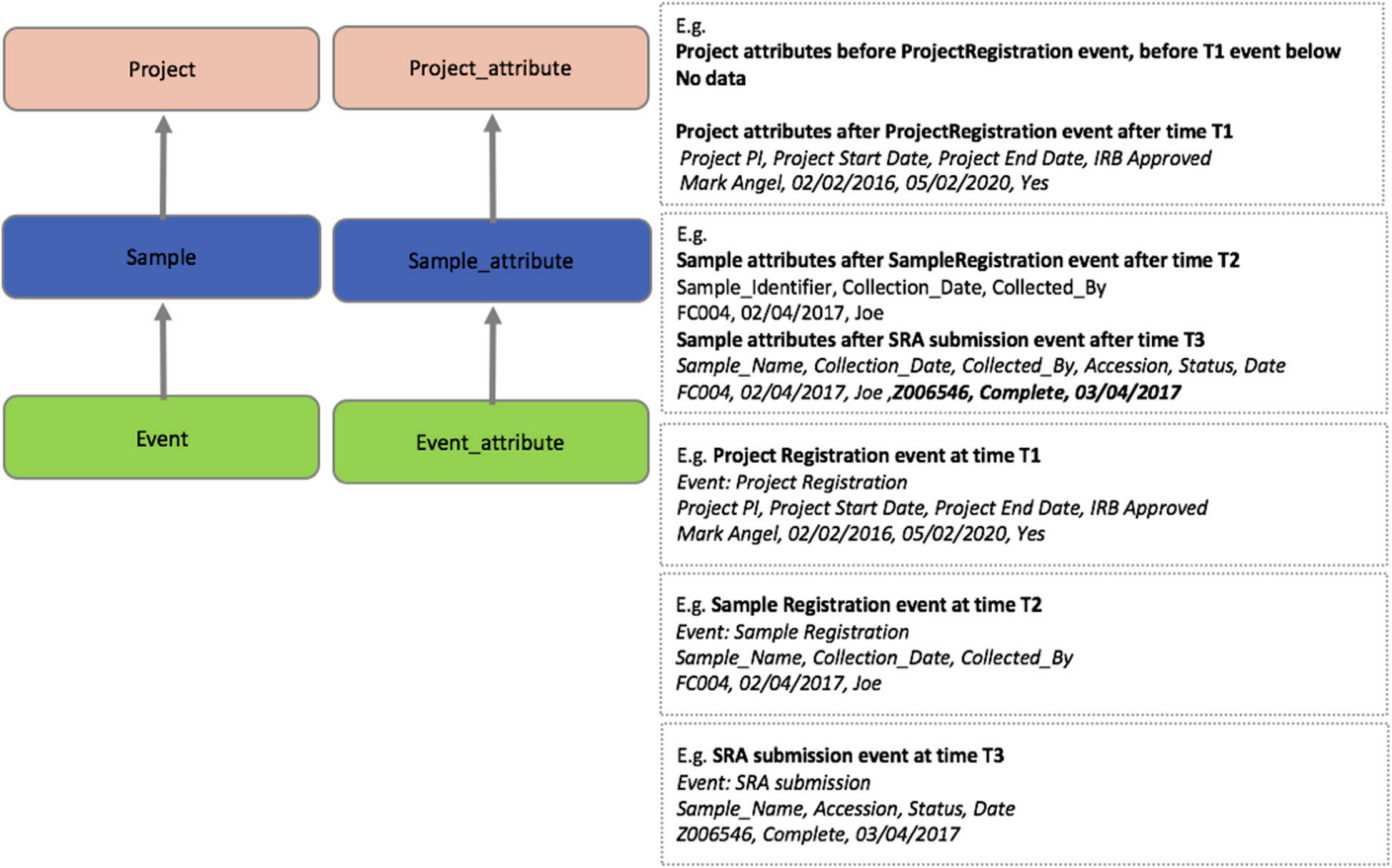
Relationship of Core Objects and Examples. The core entities of OMeta are Project, Sample, and Event. Event are defined for project or sample attributes, and after successful transaction data is stored in event, event_attribute, sample_attribute, and project_attribute table. Examples of these are in grey boxes. These represent multiple events loaded (Project Registration, Sample Registration, and SRA submission) and how data is persistent in Project_attribute and Sample_attribute entities

Project, Sample and Event entities (or tables in MySQL database terms) have core fields. Project_meta_attribute, Sample_meta_attribute, and Event_meta_attribute entities stores metadata about project, sample and event attributes, and can be customized for any fields since each field is a row, rather than a column, based on data-driven principles. Project_attribute, Sample_Attribute and Event_Attribute entities stores data after the data has been validated using event and fields defined as metadata. Relationship and examples of high-level entities and relationships are illustrated in Fig. 3.

### Security

OMeta supports project-based security. Users on specific projects can be granted “View” and “Edit” roles at the project level by the administrator. Users with “View” roles have ‘read-only’ access and may view data but cannot edit it. Users given “Edit” privileges can view and edit data stored in Ometa. The OMeta system provides complete tracking of what data is inserted or modified as well as who changed it and when, resulting in a full audit trail. All data edits are logged in event history for the audit trail. All users with access to the project can review all changes on the event history page.

### Data dictionary

OMeta has a dictionary feature that allows users to maintain large controlled lists (e.g., *species*, *genus*, and *country*). The dictionary enables field dependency, allowing for the dictionary to be set-up with a parent and client relationship. For example, if *species* is dependent on *host common name*, the dictionary can be configured so that *species* will be validated based on *host common name*.

### Integration with NCBO

OMeta has a feature to configure a metadata field with an ontology term from the NCBO [2]. If an ontology term is configured for a field, OMeta allows users to search and select for terms or subclasses in real time from Ontology. NCBO has been integrated into Ometa since it is a comprehensive open repository of biomedical ontologies that leverages the highly capable web service, REST API. Although we have integrated OMeta with NCBO, it can be integrated with any other Ontology server that employs the REST API.

### Data types

The OMeta system supports standard ‘string’, ‘date’, ‘integer’, ‘float’, and ‘file’ data types, and the data format can be applied using OMeta-provided input types or validators.

### Input types and validation

Users can configure fields as free-form ‘string’ (or text), ‘date’, ‘integer’, and numbers where only data types will be validated. Users also have the option to customize the input type style based on field input requirements. Input types can be customized into a drop-down, multi-select drop-down, checkbox, radio buttons, and datalists. Input style lets users provide allowed values in a drop-down, multi-select drop-down, radio-buttons and ontology list. Users can also customize the input type using special annotation tags. All input type annotations are enclosed in curly braces ‘{}’, followed by a keyword and the data. Below are some of the input types available for field annotation.

#### Radio button

For the *radio button* input style, the “radio” annotation keyword is used, and all radio values are enclosed in parentheses.

*{radio(Submitted;Published;Not required)}*

#### Drop-down

For the *drop-down* input style, the “dropdown” annotation keyword is used, and all drop-down values are enclosed in parentheses.

*{dropdown(Waiting for sample;Received;Sequencing;Analysis;Submitted;Completed;Deprecated)}*

#### Multi-select drop-down

The “*multi-dropdown*” annotation keyword is used to invoke the *multi-select drop-down* input style where all drop-down values are enclosed in parentheses.

*{multi-dropdown(454;Helicos;Illumina;IonTorrent;Pacific Biosciences;Sanger;SOLiD;OTH-)}*

#### Read-only

For the *read-only* input style, the “*ReadOnly*” keyword is used, followed by the default value text.

{ReadOnly:NA}

#### Regular expression-based validator

The user can specify Java regular expressions to validate data field values. To use regular expressions in Ometa, the “*RegEx*” keyword is used followed by the desired regular expression.{RegEx([ACTG]*)}

#### Custom validator

For the custom validator input style, the “*validate*” annotation keyword is used and is followed by the custom validator Java class and method name.

*{validate:DataValidator.checkFieldUniqueness}*

#### Dictionary

For the *dictionary* input dropdown, the “Dictionary” annotation keyword is used, followed by the dictionary name. The dictionary can also be set-up with parent and child relationships with cascading dependencies that allows the dependent child field to be filtered based on a selected parent field value. In the second example below, *city* list can be filtered based on the selected *state*.

{Dictionary:State}

{Dictionary:city,Parent:State}

### Web user interface

**Fig. 4:**
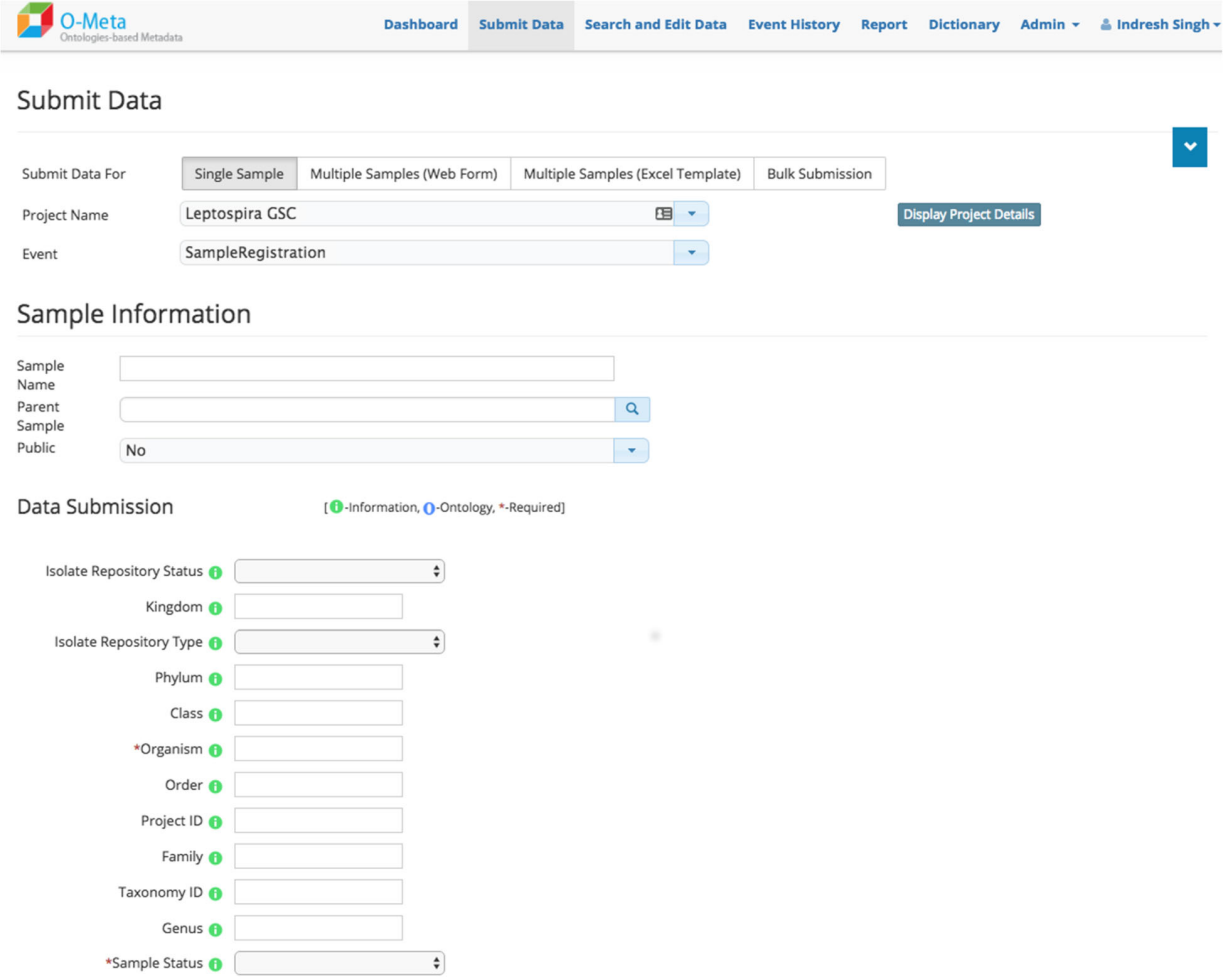
Single Sample GUI screenshot. Fields viewed on the web page are generated dynamically. These possible fields are taken from the project and event metadata configuration template. This screenshot shows an example of a Sample Registration event and fields that are configured with Sample Registration event

**Fig. 5:**
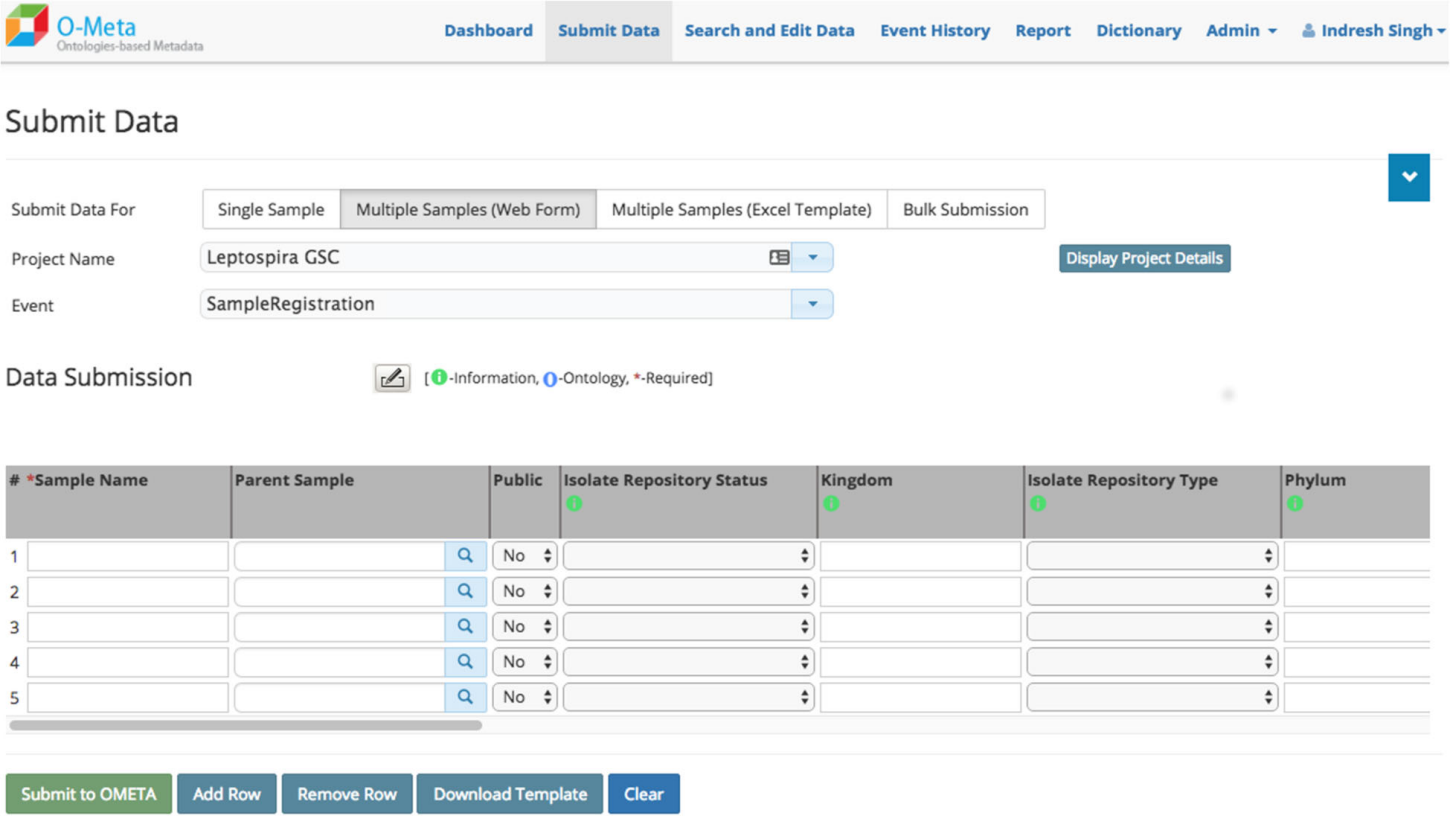
Multiple Sample GUI screenshot. Multiple sample web form allows users to enter or edit multiple samples at once rather than one sample at a time as in Fig. 4

**Fig. 6:**
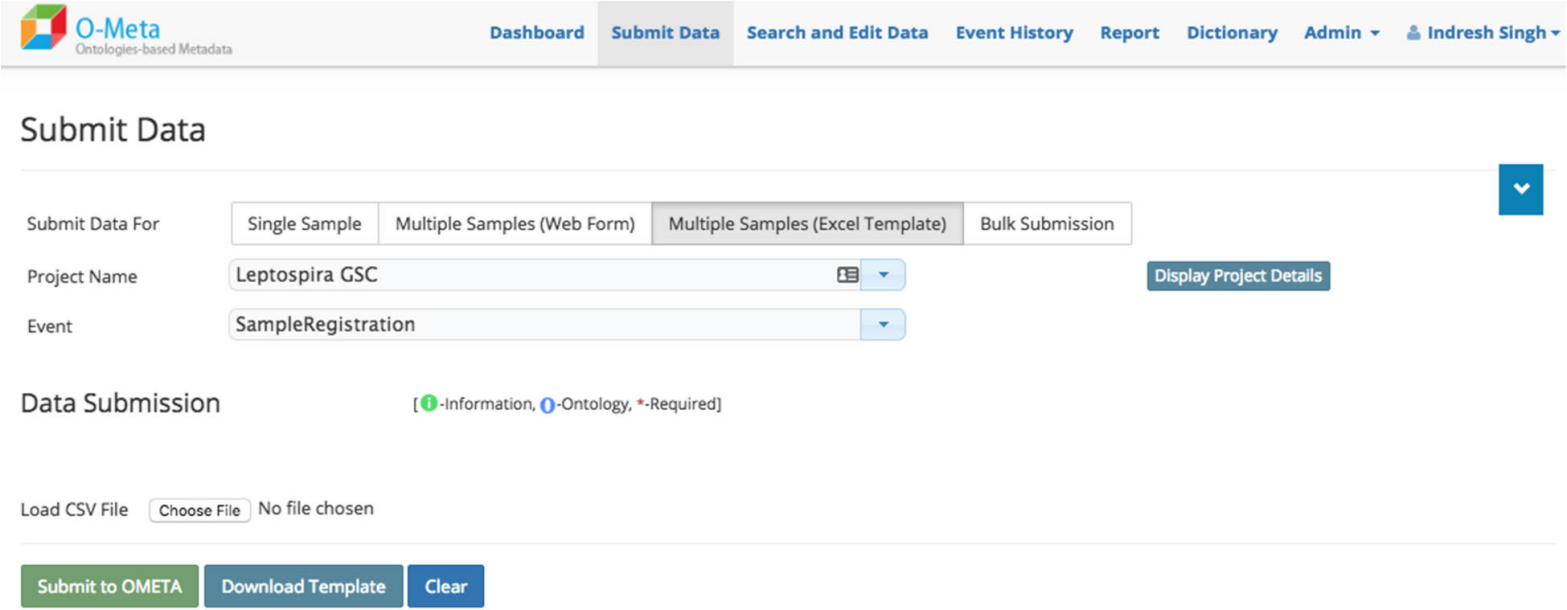
Multiple Sample Excel template file (CSV format) GUI screenshot. Interface allows users to upload of an CSV file, after upload, the web page presents data in a table format for review. The user may edit it before submission. The interface also provides a custom data standard template by selecting the “Download Template” button which users may populate and upload on this page

**Fig. 7:**
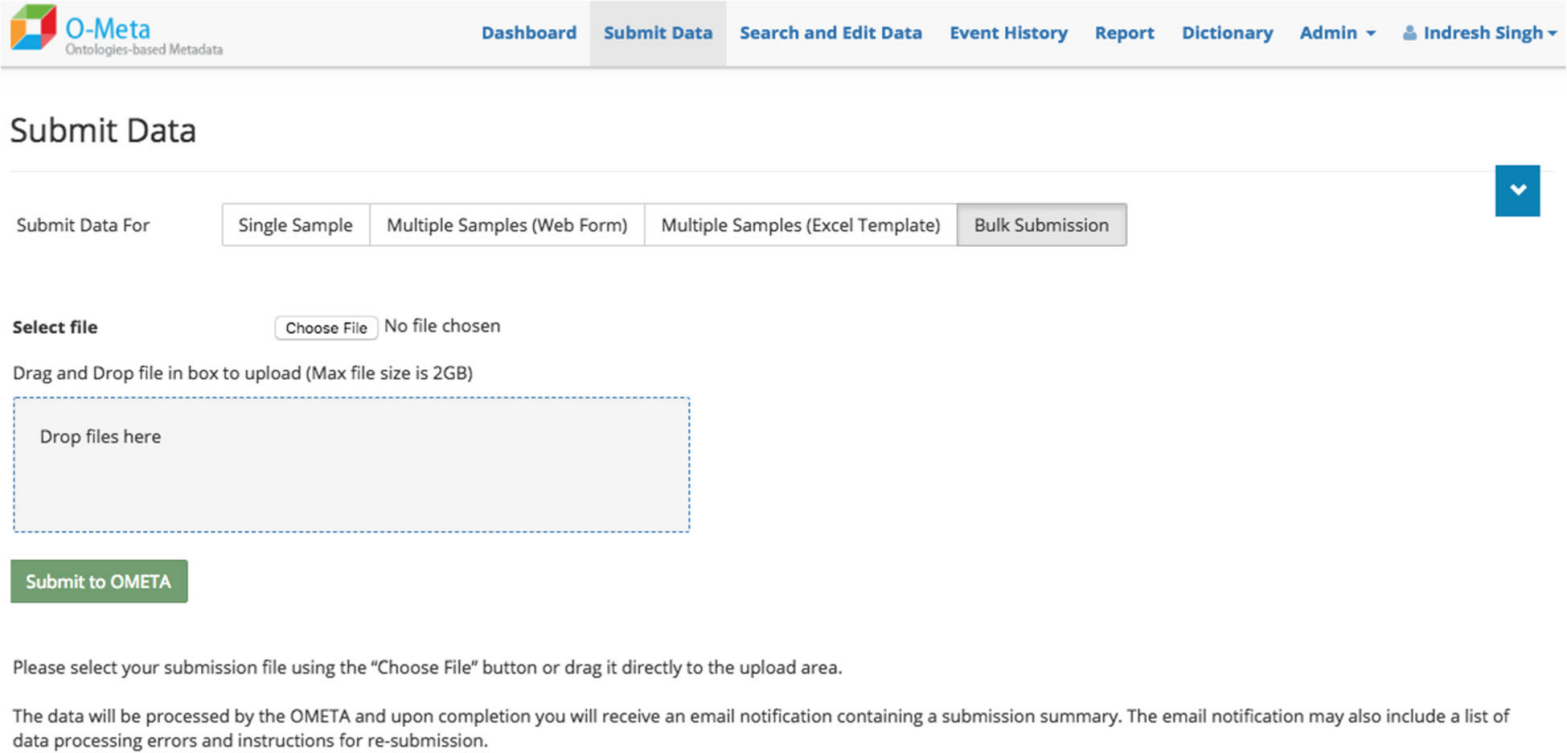
Bulk submission GUI screenshot. This page is the GUI for bulk submissions. Users may upload input files by navigating to a location of their choice, or via a simple drag-and-drop of files to the shaded grey box area. The background job scheduler processes the files and sends the user an email notification with results of successful or failed loads

**Fig. 8:**
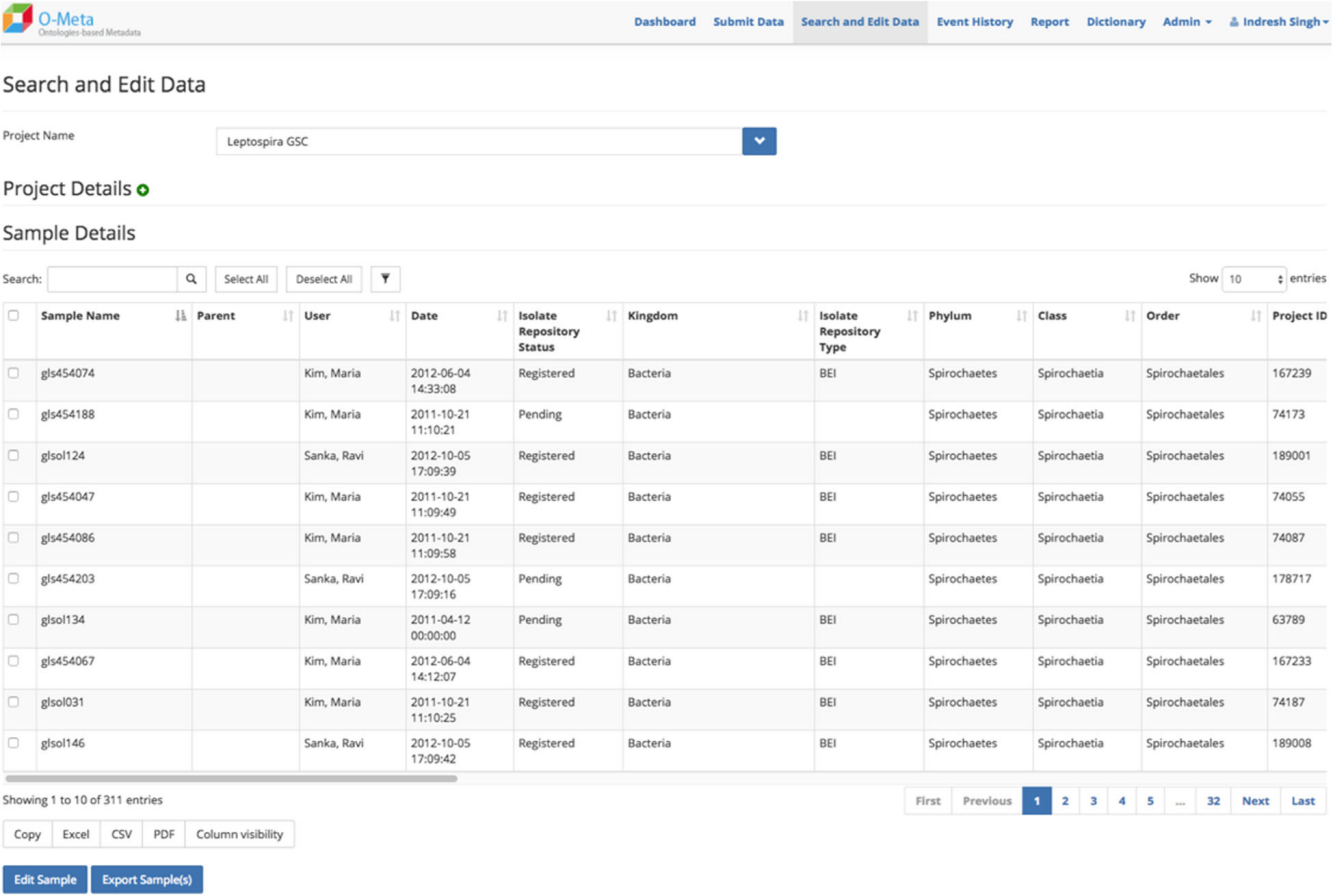
Search and Edit interface. This is a screenshot of the Search and Edit GUI. This interface allows users the capability to search and filter data. The interface supports advanced search operations such as ‘equal’, ‘like’, or ‘in’, and can join multiple fields to either expand or limit the search with Boolean operators ‘AND’, ‘OR’ or ‘NOT’

The OMeta web user interface is data-driven and dynamically generated based on the study configuration. OMeta supports a multiple user data entry interface, including the interactive and bulk interface. Users can load data via a “single sample” form (Fig. 4), an interactive “multiple samples” form (Fig. 5), a multiple sample file upload interface (Fig. 6) or a completely unsupervised bulk submission interface (Fig. 7). Users can enter one sample at a time using a simple web interface or the interactive multiple sample form. The “multiple sample” interface enables users to upload data into a single project using a standard Comma Separated Value (CSV) file. The bulk interface enables users to upload or drag-and-drop a CSV file containing all metadata as well as instructions on which project(s) and event(s) to populate. In the “bulk interface” mode, data is processed unsupervised asynchronously and processing results are sent to users via email. Below are the screenshots of all four user interfaces, all of which are generated based on metadata configured for the study. These views can be customized based on the event metadata configured for the study. OMeta has a dedicated “search and edit” web interface (Fig. 8), which provides users with the capability to search and edit data. The “search and edit” page has a “global” and “advanced” field level search capability. The advanced search tool allows users to filter data using multiple fields and supports search operations such as ‘equal’, ‘like’, or ‘in’, and joins multiple fields with Boolean operators ‘AND’, ‘OR’ or ‘NOT’. OMeta has an event history page that provides the complete audit trail of all the changes by users, including the date and time of the edits. OMeta has a report generator that can generate reports based on the event or a selected list of fields from a project or sample entity. The report can be exported in PDF or CSV format.

### Administrative interface

**Fig. 9:**
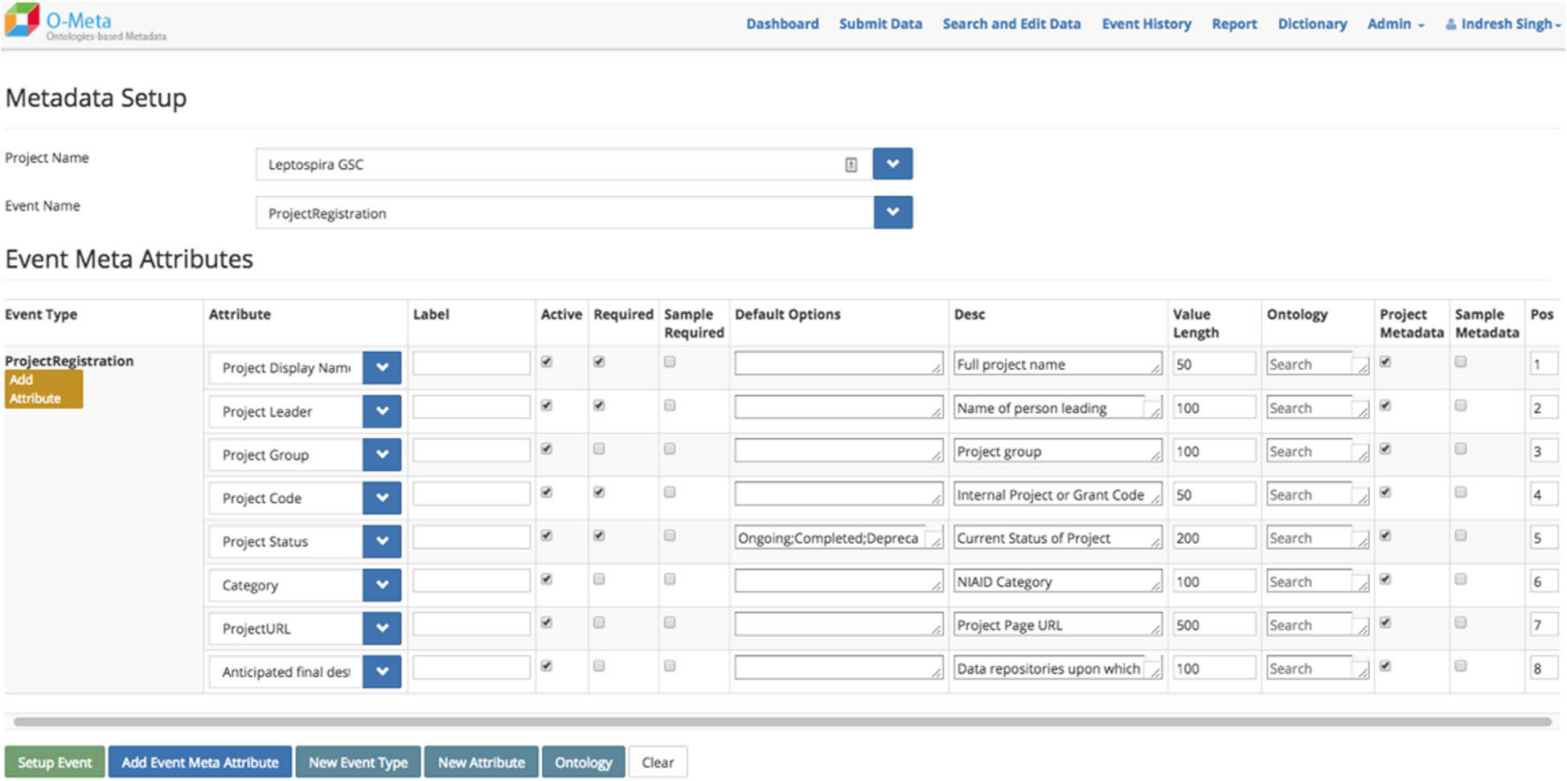
Screenshot of GUI for metadata administration page. Users who have admin privileges may add new events or customize an existing event using this metadata administration page. The page allows users with admin privileges to modify existing fields or add new fields. Users may perform actions such as mark fields as ‘active’ or they may mark them ‘inactive’ to deprecate a field. They may set whether a field is required or optional, set the input style in default options, set field description, set max field length, set ontology class and set field position on the event page

The OMeta “administrative” interface allows for the management of project registration, project metadata setup, user, user roles, project roles, dictionary management, and JSON export management. The project metadata set-up page (Fig. 9) allows an administrator to quickly set up and update events and metadata based on study design. Project metadata can also be configured or updated using a command line interface (CLI) (see below). The JSON export management page allows an administrator to set-up and schedule predefined jobs to export data in JSON format. JSON is a lightweight data-interchange format that can either be used for data integration in other applications or as a simple data export. The JSON exporter allows users to select a project and the fields from project or sample metadata for export.

#### Federated integrated systems

Federated integrated systems allows interoperability and information sharing between different systems. The OMeta system has features that can be integrated with other OMeta instances or other systems using secure remote EJB calls and REST APIs. We are planning to provide REST APIs to query all data types to fully support system integrations across multiple systems.

#### Command line interface (CLI)

OMeta provides support for users to load and query data using a CLI in addition to the graphical user interface (GUI). It also enables users to configure a study and customize metadata for new studies from simple CSV files. Below is an example of CLI loading command using a data file named samples.csv. Basic examples of project and sample registration setup for GSC/BRC Metadata Standards and MIxS-human gut data standards are provided in the Additional files 1, 2, 3 and 4.

*$*. /*load_event.sh HMP SampleRegistration samples.csv*

*Sample.csv* (data should be in CSV format but for better presentation it is presented here as a Table 1)

**Table 1:** Sample Registration Template. Data should be in CSV format but for better presentation it is presented here as a table. CSV file starts with template name on first line, field headers are on second line, and data rows afterwards

#DataTemplate: Sample Registration						
Project Name	Sample Name	Sample Status	Sample Type	Organism	Specimen Collection Date	Specimen Collector Name
Pilot647	SAM657	Analysis	GDNA	*E. coli*	7/27/16	John Kim
Pilot647	SAM657	Analysis	CDNA	*E. coli*	10/20/14	Ted Michael

## Use case 1: metagenomics

### Background

OMeta’s inherent flexibility lends itself to use with various types of projects. Here we present a use case example of a metagenomics study. This implementation of OMeta was for the management and tracking of a large dataset of young twins in an oral microbiome study (R01DE019665) whose participants were recruited from Australia between 2014 and 2016 [3, 4]. The study was comprised of 2310 oral biofilm samples from 1011 twin subjects. These samples went through varying stages of nucleic acid extraction, library preparation for sequencing, sequencing, and data analysis. The complexity of this large study required a tool for accurately tracking thousands of samples through the system. The ability to record the status of the sample, such as the time of sample receipt or the stage of sample laboratory processing (e.g., nucleic acid extraction, sequencing, etc.) was crucial for efficient/reliable sample management at this scale. OMeta allowed users to record the physical and clinical metadata for each sample.

### Study metadata standards

The flexibility of the OMeta platform comes from its ability to provide users with the capability to fully customize the metadata standards and data fields (Fig. 2) to address the specific needs of the individual study. For the oral twin study, the metadata format template was based on the MIxS/MIMS standards [5] proposed by the Genomic Standards Consortium (GSC) [6, 7]. Some data fields from the basic MIMS standard were omitted where it was not needed (e.g., temperature, salinity, pulse) and other data fields were added to the metadata format standards template where the MIMS standards did not address specific project metadata requirements (e.g., zygosity, twin_ID). OMeta’s flexibility allows customization of the study metadata standards template without code change to successfully meet the project needs.

### Data transformation

Since OMeta utilizes CSV text files as input for loading sample information into the database, writing software for parsing raw text files into the requisite CSV format for import into OMeta is a straightforward task. Physical and clinical metadata were collected by collaborators at two different clinical sites in Australia and delivered to the JCVI. One collaborating group delivered Excel™ spreadsheets, while the other group delivered data dumps from their own proprietary database. In both cases, metadata was converted to tab-delimited text files and readily passed through the parser. The parsing software translated the extracted text files into CSV input files ready for upload to OMeta.

### Validation and sample tracking

Inherent in OMeta’s design are comprehensive validation methods that ensure sample integrity. For example, the platform verifies that the entries are unique and will issue warnings if any entry violates the validation constraints. As a part of the upload process, OMeta timestamps each sample entry and attaches user information for tracking and audit purposes. No transaction takes place without a record of the process - who it was performed by and when it occurred. Any failed transactions are rollback to maintain the integrity of data.

### Management/administration

Management and administration of the application was straightforward. OMeta allowed controlled access of the application by project and application roles. Any user can be given anything from full administrative privileges to simple view and edit access roles on selected projects. Application administrative roles allowed users to set-up new users or customize project metadata fields or controlled vocabulary. Since the platform is web-based, users can access the database from anywhere in the world with any web browser making it operating system agnostic. Collaborators from the University of Adelaide in Adelaide, Australia as well as from the Murdoch Children’s Research Institute in Melbourne, Australia were granted access to the Ometa database for the project. JCVI has a physical presence on the east coast of the United States in Rockville, MD, and on the west coast in La Jolla, CA. Individual users at all four locations required access to the database fo uploads, review and information retrieval.

### Custom queries and reports

OMeta has an interface that enables custom queries of the database. All users with access to the database can make simple or complex queries to retrieve data. These data can be exported in different document formats for use in downstream data analyses or for submission of metadata for BioSample registrations at NCBI/GenBank. The project involved different submissions of sequencing data as well as the corresponding metadata to GenBank. Queries could be performed to generate reports of all physical and clinical metadata for a specific subset of twin subjects for the express purpose of generating the requisite files GenBank requires for BioSample registrations. Reports could also be generated for creating data files for use in analyses such as statistical hypothesis testing. Reports could be easily modified and then uploaded into statistical analysis software packages such as R [8] .

### Metagenomics use case summary

The OMeta platform has proven to be a very flexible and capable tool for sample tracking of a large metagenomics study. Once the project and its metadata were configured, the tracking of multiple samples from multiple subjects was easier. The sheer number of samples delivered from different collaborators, from different subjects, collected over the course of 18 months would have been difficult to manage. OMeta made the process more manageable.

## Use case 2: whole genome sequencing (WGS) studies

### Background

The JCVI Genomic Center for Infectious Diseases (GCID) (U19AI110819) and previous contract Genomic Sequencing Center for Infectious Diseases (GSCID) (HHSN272200900007C) were established by the NIAID to develop basic knowledge of infectious disease biology through the application of DNA sequencing, genotyping, and comparative genomic analysis. The goal of the JCVI GCID is the application of innovative genomics-based approaches to study pathogens and determinants of their virulence, drug-resistance, immune evasion, and interactions with the host and the host microbiome to advance research in pathogenicity, drug-resistance, disease transmission, and vaccine development. The GCID and GSCID contracts have multiple studies and samples encompassing thousands of isolates of bacterial, fungal and parasitic organisms. Each study was/is unique with different goals and metadata requirements, thus requiring customization of the isolation methods, metadata, and analysis. The GCID/GSCID contract has 110 studies with 5972 samples and 156,675 sample attributes across bacterial, fungal, and parasite projects. We started with creating and configuring custom databases for each individual GCID project. As the number of projects increased, we encountered challenges of keeping metadata standards and metadata harmonized with evolving metadata tracking and validation requirements.

In 2013, we surveyed open source tools available for metadata tracking (see Discussion), including the ISA tool. Although there are many data standards, there are very few tools to manage data standards and manage data. The ISA tool is a flexible tool that provides metadata tracking based on standards and provides flexibility to configure and extend the metadata. However, the ISA tool does not provide centralized data management with an audit trail of all changes, and that is a key shortcoming since it is one of the core requirements for centralized metadata tracking.

### Metadata standards and schema

For the GCID, we started configuring OMeta based on specified study goals and metadata requirements. In 2014, GSCID/BRC Project and Sample Application Standard [9], developed by representatives of the GSCIDs, the BRCs for Infectious Diseases, and the NIAID, part of the National Institutes of Health (NIH) was published. The data standards were designed to capture standardized human pathogen and vector sequencing metadata to support epidemiologic and genotype-phenotype association studies for human infectious diseases. The GCID consortium adopted the GSCID/BRC Project and Sample Application Standard, and JCVI team implemented this standard in OMeta. OMeta’s flexibility also enabled us to add additional fields for internal tracking like sample status, comments, assembler, assembly coverage, short read archive (SRA) submission status, SRA submission date, GenBank submission date, GenBank accession, etc. For the GCID, we prepared an Excel™ sheet template based on GSCID/BRC standards to collect and exchange data with our collaborators and other researchers.

### Metadata tracking, validation, and transformation

All collaborators who provided samples were required to collect and submit metadata in a GCID Excel™ metadata sheet. Metadata from a GCID Excel™ sheet was converted to CSV file format and uploaded into OMeta. During the uploading process, additional data validation checks were performed to check for data integrity and proper data format. Data integrity checks like valid date, unique sample name, checks for required fields for NCBI BioSample submissions (e.g., latitude and longitude), checks for valid data from controlled vocabulary were also implemented. Error reports were generated for fields that did not comply with data standards. As part of the uploading and tracking process, OMeta maintained timestamps and user information - components which provide critical information such as what has changed, when it changed, and who was responsible for the changes.

OMeta allows multiple, incremental changes/updates to any record. We have updated the data in OMeta various times, such as after sequencing, assembly, annotation, delivery to SRA, and GenBank submission. After sequencing, we updated the status of the sample to record cases where there may be failures due to library preparation, sequencing or contamination. If the sample was contaminated, the sample was deprecated and removed from further analysis. After assembly, OMeta was updated with the name of the assembler used as well as any relevant assembly statistics. After annotation, delivery to SRA and Genbank submission, OMeta was updated with status and accession IDs provided by SRA and GenBank for tracking and further downstream analysis.

OMeta’s easy to use web-based interface allowed researchers, collaborators, and lab technicians to load, view, edit or export data from anywhere in the world with no knowledge of the behind-the-scenes inner workings of the database.

### Project level security and management Interface

OMeta provided an easy interface for setting-up new users and set-up for project level access to those users. OMeta provided read-only and edit roles that allowed us to control who could view and edit data but all GCID projects were public and read-only access was granted to all registered users. The template management interface allowed us to customize the values for the fields as required by each individual study.

### Reports and export data

OMeta has a reporting interface which allows users to view reports based on existing data standards, and also provides an easy interface for creating new reports by using metadata fields available in the study. Reports could be exported in different document formats such as CSV, Portable Document Format (PDF), or Excel™ spreadsheets. Advanced users or developers could also generate reports directly accessing the database via queries. Data could be exported in CSV format and could be used for downstream data analyses or integration. For the GCID project, data exported from OMeta was used for BioSample registration at GenBank, or submission to PATRIC [10]; generation of configuration files to label phylogenetic trees (e.g., “isolation date”, “isolation source”:, “isolation location”); and pan-genome “groups” analysis (i.e., metadata to genotype associations) - to identify genes and flexible genomic islands shared by isolates within one metadata group, but absent from other metadata group(s). Data exported in CSV format was also used for editing the data offline and resubmitting back to OMeta to update the data.

### WGS use case summary

The OMeta platform has proven to be an easy to use, flexible tool for developing templates for recording and validating metadata, and sample tracking for large whole genome sequencing studies. Once the study’s metadata was designed and configured, OMeta allowed us to easily create new studies using the existing studies as templates. We have successfully tracked 110 studies with 5972 samples and 156,675 sample attributes across bacterial, fungal, and parasite projects. OMeta provided a very flexible interface for managing and customizing templates for recording metadata, tracking, and exporting data for data exchange with other data banks and bioinformatics resource centers such as NCBI, PATRIC [10] or ToxoDB [10, 11].

## Discussion

Large genomics studies often involve the collaboration of multidisciplinary researchers utilizing several high-throughput omics platforms. These studies include different sample types, experiments, assays, and analysis methods requiring multiple data standards and ontologies. There are many data standards and ontologies; the Genomic Standards Consortium’s minimal information (MIxS) standards, NCBI’s BioSample metadata standards, GSCID/BRC Project and Sample Application Standard, DMID Clinical Metadata Standards, Cancer Data Standards Registry and Repository (caDSR), CDISC, BioAssay Ontology, Environment Ontology, Mass Spectrometry Ontology, Ontology for Biomedical Investigations (OBI), Chemical Information Ontology, Cell Ontology. Currently, the NCBO ontology bioportal contains 843 biomedical ontologies. Even with these data standards and ontologies, most of the studies require customization to better ‘fit’ the metadata due to the novel and evolving nature of research. We evaluated several leading, existing open source tools. None of the tools provided all the necessary functionality and flexibility required for our uses, necessitating creation of OMeta. OMeta has been used by multiple studies and center projects like GSCID/GCID, JCVI Human Microbiome Project (HMP) and Data Processing and Coordinating Center (DPCC) of the NIAID Centers of Excellence for Influenza Research and Surveillance (CEIRS).

The OMeta tool has been adopted and customized by the DPCC [12]. The DPCC supports the data management needs of five CEIRS centers; Center for Research on Influenza Pathogenesis (CRIP), Emory-UGA Center of Excellence for Influenza Research and Surveillance, Johns Hopkins Center of Excellence for Influenza Research and Surveillance, New York Influenza Center of Excellence (NYICE), and St. Jude Center of Excellence for Influenza Research and Surveillance. The CEIRS DPCC has implemented 17 data standards templates across surveillance, serology, viral isolate, sequencing assays and reagents to collect, curate and manage metadata.

Table 2 provides a comparison of critical and unique features of OMeta with some of the existing tools for tracking metadata. Only OMeta provided comprehensive event based metadata management and a complete audit trail.

**Table 2:** Comparison of metadata tracking tools

Features/Tools	OMeta	ISA-Tool	LabKey	CKAN	XperimentR	ICAT
Event based tracking	Yes	No	No^a^	No	No	No
Open-source Project	Yes	Yes	Yes	Yes	Yes	Yes
Project Role-Based-Security	Yes	No	Yes	Yes	Yes	Yes
Ontology Integration & Lookup	Yes	Yes	No	No	Yes	No
Configurable metadata	Yes	Yes	Yes	No	Yes	Yes
Configurable validation	Yes	No	No	No	No	No
Web Interface	Yes	No^b^	Yes	Yes	Yes	Yes
Complete Audit trail	Yes	No	No	No	No	No
Asynchronous data loading	Yes	No	Yes	No	No	Yes
Row-level bulk data loading	Yes	No	No	No	No	Yes
HIPAA compliance	No	No	No^c^	No	No	No
Built-in LDAP Authentication	Yes	No	Yes	Yes	Yes	Yes

^a^Labkey has a process to set-up and load data, but it is not a true event-based system

^b^ISA-Tool has a ISA-creator that is Java application that can run on a desktop computer, but it lacks the web interface for creating, editing and managing data

^c^LabKey Community Edition is not HIPAA compliant, but LabKey Enterprise Edition has HIPAA compliant features

### ISA software suite

The ISA software suite [13] is an open source software suite that provides metadata tracking and provides tools for metadata customization, validation, ontology look-up, semantic representation in Resource Description Framework (RDF) format, import, and export capability. The ISA suite is widely used to collect, curate, and exchange data, but we did not adopt ISA suite since it does not have some of the critical features for centralized metadata management that we needed such as a web interface to collect, curate or exchange data, event-based or process-based tracking, history of changes or audit trail, and flexible real-time reporting.

### LabKey

LabKey [14] is an open source tool for scientific data integration, analysis, and collaboration including data management, specimen management and lab process tacking. LabKey provides extensive features for metadata management, and it has easy to use wizard driven user interface to import, export and search data. It has been adopted and customized by scientific and research communities, but LabKey has a steep learning curve and requires a fair amount of coding to implement new data standards and validations. LabKey is a good option to fulfill the requirements for a comprehensive system that provides metadata management and lab process tracking, but we did not adopt a LabKey framework as it failed to provide a data-driven framework, one of the key requirements for metadata tracking tool.

### CKAN

CKAN [15] is an open source tool for making open data websites. Although it allows users to load data in multiple formats and provides efficient search features, it does not have any functionality to configure metadata standards, validate data during loading, or provide a history of changes to the data. CKAN provides a good way to aggregate and search the data, but it does not provide the required functionality for metadata management.

### XperimentR

XperimentR [16] is a web-based open source application for laboratory scientists to capture and share experimental metadata. XperimentR uses the ISA-tab data model and has features to configure, store and export metadata with an experiment, but its primary focus is to track and annotate the lab process. Although XperimentR is a good tool for basic metadata and lab process tracking, it did not provide us with a flexible way to set-up the metadata standards and provide a history of all the changes in metadata.

### ICAT

ICAT [17] is an open source metadata catalog tool with a flexible and extensible architecture designed to support experimental data from large research facilities. ICAT is built on a core scientific metadata model (CSMD) developed by the Science & Technology facilities Council (STFC) and has several components including the ICAT server, ICAT manager, ICAT client, and the ICAT data service. ICAT provides a good API but does not provide a web-user interface to collect, curate and validate data. Furthermore, it lacks the concept of metadata standards, templates, and validation of metadata based on metadata standards.

### Limitations and lessons learned

#### File formats support

OMeta supports metadata and data ingestion, import or export in CSV file format only. Data files may be attached in any other format, but the metadata file must be formatted as a CSV file.

#### Multi-hierarchy metadata

OMeta supports sample hierarchy using parent-client relationships but does not support multi-hierarchical objects as part of the metadata. We plan to extend OMeta to support JSON file format in order for OMeta to be able to support multi-level object hierarchies and efficient dependency tracking between fields.

#### Dictionary

Although the dictionary feature currently only allows for the selection of one value, it can be easily extended to support multiple values. In a future release, we will make enhancements to allow the user-determined dictionary to be a part of other drop-down and multi-selected drop-down modifiers.

#### Application query performance

OMeta was designed with data-driven principles to be flexible and agile because metadata is a very small fraction of all data. For one of the larger projects, we loaded greater than 500,000 samples with total attribute counts of greater than 17 million. Most of the functionality worked as expected, but the data export page timed-out due to processing time to fulfill the query and packaging the resultant data into a zip archive file. The same export query performed on the CLI worked as expected. OMeta is making architectural changes to support large exports by making it an asynchronous job.

### Future directions

#### Support for ISA-tab format and integration

ISA-tab is widely used in the genomics community and ISA software tools provide viewing and editing features in ISA-tab format. We are planning to add support for ISA-tab format to allow for the user community to view, edit and submit data in ISA-tab format. This feature will allow the ISA community to use OMeta as their centralized metadata tracking system with extended features.

#### OMeta indexing

The OMeta team is working on adding Apache Solr indexing to support enterprise level efficient and scalable data search capabilities. Apache Solr is a standalone enterprise search server with a REST-like API that provides highly scalable indexing and searching capability of JSON, XML, CSV or binary over HyperText Transfer Protocol (HTTP).

#### OMeta persistence storage

Although OMeta has been using relational data tables in MySQL, we are also exploring options to store objects as JSON objects for efficient storage and retrieval. We are also exploring options for using MongoDB as the database. MongoDB is an open-source, non-relational database developed by MongoDB, Inc. MongoDB stores data as documents in a binary representation called BSON (Binary JSON). MongoDB has the advantage of permitting fast queries since all fields related to an object are stored as a document, and it provides the ability to represent hierarchical relationships to easily store arrays and other more complex structures.

#### Visualization using graph database

We are exploring graph database for metadata visualization [18] for showing clustering and relationship between samples.

#### Scripting

We intend to add scripting capability for users to be able to integrate and incorporate JavaScript and R script as part of the tool for analysis and visualization.

#### Virtualization using Docker

Application virtualization technology, Docker [19] is a platform designed to make it easier for an application developer to create, deploy, distribute and customize an application by using containers [20]. Docker containers are based on open standards and run on all major platforms Linux, Microsoft Windows, Apple macOS, or any infrastructure including VMs, and in the cloud. We intend to build and provide a Docker container image for the research community for easy deployment and integration.

## Conclusions

The scientific research community recognizes the importance and necessity of standards and metadata collection for biological samples and experiments as they pertain to fundamental research. Although there are many data standards and ontologies to support these needs, there is no data-driven flexible tool that can be quickly configured as studies and analysis processes evolve. The OMeta metadata tracking system builds on data-driven principles to fill this gap and facilitates data standards compliance by providing an intuitive platform for the configuration, collection, curation, visualization, storage, and sharing of metadata.

## Additional files

Additional file 1: GSC/BRC Metadata Standards ProjectSetup: Example CSV file for setting up Project registration and update event for GSC/BRC metadata standards. User as load the setup file using CLI interface or user can setup project using metadata setup GUI. (CSV 14 kb)

Additional file 2: GSC/BRC Metadata Standards SampleSetup: Example CSV file for setting up Sample registration and update events for GSC/BRC metadata standards. (CSV 61 kb)

Additional file 3: MixS human Gut Data Standard ProjectSetup. Example CSV file for setting up project registration and update events for MixS human Gut Data Standard. (CSV 7 kb)

Additional file 4: MixS human Gut Data Standard_SampleSequencingAssaySetup. Example CSV file for setting up sample registration and update events for MixS human Gut Data Standard. (CSV 19 kb)

## Abbreviations

API: Application programming interface
BRC: Bioinformatics Resource Centers
CEIRS: Centers of Excellence for Influenza Research and Surveillance
CLI: Command line interface
CRIP: Center for Research on Influenza Pathogenesis
CSMD: Core scientific metadata model
CSV: Comma separated values
DPCC: Data Processing and Coordinating Center
GCID: Genomic Center for Infectious Diseases
GSC: Genome Sequencing consortium
GUI: Graphic User Interface
HMP: Human Microbiome Project
HTTP: HyperText Transfer Protocol
JCVI: J. Craig Venter Institute
JSON: JavaScript Object Notation
LDAP: Lightweight Directory Access Protocol
MIMS: Minimal Information Metagenomic Sequence/Sample
MIxS: Minimal Information about any (x) Sequence/Sample
NCBI: National Center for Biotechnology Information
NCBO: National Center for Biomedical Ontology
NIAID: National Institute of Allergy and Infectious Diseases
NYICE: New York Influenza Center of Excellence
OBI: Ontology of Biomedical Investigations
PDF: Portable Document Format
RDF: Resource Description Framework
REST: REpresentational State Transfer
STFC: Science & Technology Facilities Council
VM: Virtual Machine
